# The Soybean Basic Helix-Loop-Helix Transcription Factor ORG3-Like Enhances Cadmium Tolerance via Increased Iron and Reduced Cadmium Uptake and Transport from Roots to Shoots

**DOI:** 10.3389/fpls.2017.01098

**Published:** 2017-06-28

**Authors:** Zhaolong Xu, Xiaoqing Liu, Xiaolan He, Ling Xu, Yihong Huang, Hongbo Shao, Dayong Zhang, Boping Tang, Hongxiang Ma

**Affiliations:** ^1^Salt-Soil Agricultural Center, Institute of Agricultural Resources and Environment, Jiangsu Academy of Agricultural SciencesNanjing, China; ^2^JLCBE, Yancheng Teachers UniversityYancheng, China; ^3^Institute of Grain Crops, Jiangsu Academy of Agricultural SciencesNanjing, China

**Keywords:** soybean, bHLH transcription factor, *GmORG3*, cadmium tolerance, gene expression

## Abstract

Cadmium (Cd) is one of the most dangerous heavy metal pollutants in the environment and is toxic to animal and plant cells. On the other hand, iron (Fe) is an essential element for plant growth and development. The chlorosis of plant leaves under cadmium stress is similar to the typical symptom of iron deficiency. Recently, several *Arabidopsis* basic/helix-loop-helix (bHLH) transcription factors have been identified that are involved in the interactions between Cd and Fe. In the present study, over-expression the ORG3-like gene *GmORG3*, a bHLH transcription factor OBP3-responsive gene (*ORG*), enhanced Cd tolerance and stabilized Fe homeostasis. The domain analysis of GmORG3 showed that the protein contains a conserved 61-residue bHLH domain belonging to subfamily II. Moreover, subcellular localization experiments showed that GmORG3 is a nucleoprotein. *GmORG3* was transcribed only in soybean roots and was significantly induced by external Cd stress in soybean plants. Heterologous expression of *GmORG3* enhanced Cd tolerance in yeast. Furthermore, the overexpression of *GmORG3* in soybean mosaic seedlings using a hairy root system showed that overexpressing plants increased the translocation ratio of Fe but reduced Cd translocation from the roots to shoots. In addition, the ectopic expression of *GmORG3* in tobacco reduced phytotoxic effects induced by Cd stress and Fe deficiency, including the blockage of root elongation and decreased chlorophyll content. By integrating all these results, we found that *GmORG3* plays an important role in response to Cd stress. The results provide new insights into the molecular mechanisms of Cd tolerance in soybean.

## Introduction

Soil heavy metal pollution is increasingly becoming a global environmental problem. Generally, heavy metals are naturally occurring in the soil, however, their amounts also increase due to human activities. Community development activities are causing the rapid expansion of non-agricultural construction and wastelands, and farming activities such as the increased use of agricultural fertilizers, especially phosphate fertilizer, as well as sewage sludge, wastewater, and pesticides are leading to soil heavy metal pollution. And these heavy metals may be absorbed by crops, thus enter the human body (Puga et al., 2015).

Direct or indirect human interference is continuously increasing levels of heavy metals (Larue et al., 2010; Huang et al., 2011), thus, the effects of this interference on soil occur worldwide. Cadmium (Cd) is a harmful heavy metal element and has become a serious problem for human health. Because of high water solubility, Cd is considered to be one of the most toxic heavy metal in the nature world. Even at very small concentrations, it is strong toxic to living organisms, and it is the fourth strength toxic heavy metal element to vascular plants, exists in soils as well (Huang et al., 2011; Xie et al., 2013; Pietrini et al., 2015). The concentrations of Cd as little as 8 mg/kg in the soil are toxic to plants (Pedro et al., 2013). Plants growing under cadmium stress exhibit leaf etiolation and arrested root elongation and can die (Kahle, 1993; Das et al., 1997). Cd gets into plant cells through Fe, zinc (Zn) and some other transporters/channels. There are several mechanisms are developed in plants for Cd detoxification, such as binding in cell wall, compartmentation in vacuole, chelation with phytochelatins, and enrichment in leaf epidermal hairs (Clemens, 2006).

Iron (Fe) is one of seven indispensable trace elements needed for plant growth. Iron deficiency is common in plants and animals (López-Millán et al., 2013; Boonyaves et al., 2017). Iron deficiency in plants is generally a problem of iron availability, rather than the supply of iron, because iron is the fourth highest rich element on the surface of the globe. Most of the plants can uptake and make use of soluble ferrous iron (Fe^2+^) that is ubiquitous in soils with the pH between 6.5 and 7.5 (Havlin et al., 2005). However, under alkaline conditions (pH > 8), Fe^3+^ in the soil is in an insoluble state that can’t dissolve in water, and this Fe^3+^ is difficult to be absorbed by most plant. Therefore, not enough Fe is introduced into food chain. In developing countries, most of the 0 to 10-year-old children and pregnant women (at least 30–40%) suffer from a lack of iron^1^. Anemia is used as an indirect indicator. Globally, anemia affects approximately 2 billion people or approximately 25% of the population (WHO, 2016). Iron deficiency chlorosis in plants caused a losses of about $120 million annually in the United States alone (Hansen et al., 2003). Therefore, there is a need to improve Fe uptake by plants to introduce Fe into the food chain.

The basic helix-loop-helix (bHLH) transcription factors compose a superfamily and have been studied comprehensively in eukaryotic organism cells, especially in mammalian cells, in which the genes expression, protein structure, function, and phylogenetic analyses have been thoroughly and comprehensively performed (Ledent and Vervoort, 2001; Heim et al., 2003; Toledo-Ortiz et al., 2003). The research data demonstrate that the bHLH transcription factors are a type of significant regulatory component in gene expressional networks in organisms, involved in a wide variety of processes, such as cell proliferation, differentiation and responses to abiotic stress (Grandori et al., 2000; Massari and Murre, 2000; Yuan et al., 2008; Wu et al., 2012). Wu et al. (2012) reported that AtbHLH38 and AtbHLH39 interact with AtbHLH29 to enhance the Cd tolerance of *Arabidopsis* seedlings via decreased cadmium transfer from roots to shoots and to improve the iron homeostasis and concentration of shoots. The data also showed that the overexpression of AtbHLH39 alone can improve the capability of Cd tolerance in the seedlings. The transcription factor AtbHLH29 is critical to the regulation of iron uptake (Colangelo and Guerinot, 2004; Jakoby et al., 2004; Yuan et al., 2005). Under Fe deficiency, the expression of *AtbHLH100* and *AtbHLH101* is up-regulated in roots and leaves (Wang et al., 2007). The rice bHLH gene *OsIRO2*, which is orthologous to *AtbHLH38* and *AtbHLH39*, is an essential regulator of the genes involved in Fe uptake under Fe deficiency conditions (Ogo et al., 2007).

In this study, we investigated the functions of a soybean bHLH transcription factor GmORG3 regarding Cd tolerance and Fe uptake. *GmORG3* is an ortholog of *AtbHLH39*, which is involved in the absorption and translocation of Cd and Fe. The overexpression of *GmORG3* in yeast enhanced yeast cell tolerance to Cd, and the overexpression of GmORG3 in soybean roots of mosaic seedlings reduced the translocation ratio of Cd from roots to shoots. *GmORG3* transgenic tobacco plants were more tolerant than wild-type plants were under Fe deficiency.

## Results

### Phylogenetic Analysis of GmbHLH TFs and the Domain Analysis and Subcellular Localization of the GmORG3 Protein

**FIGURE 1:**
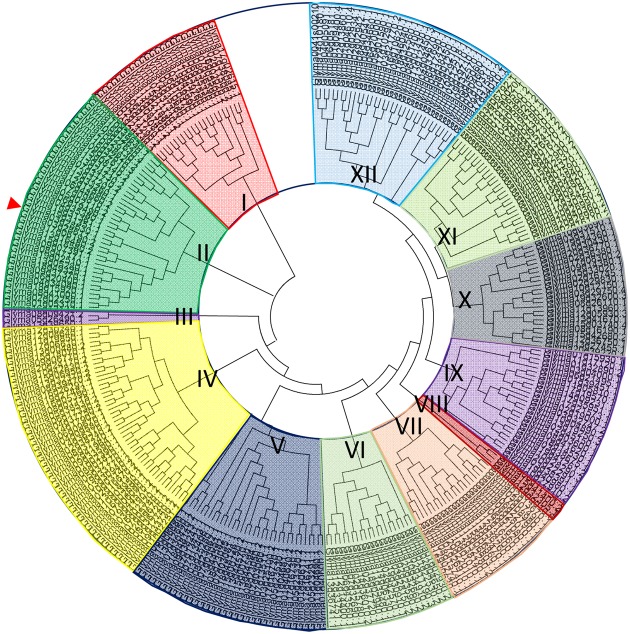
Phylogenetic analysis of GmbHLH TFs. Phylogenetic tree containing 308 full-length soybean bHLHs; GmORG3 is indicated by the arrow. Multiple sequence alignment containing the amino acid sequences of 308 bHLH proteins was performed using ClustalX2. The phylogenetic tree was constructed using the neighbor-joining method with MEGA software. The bHLH transcription factors were divided into 12 subfamilies titled I–XII. GmORG3 belonged to subfamily II.

There are 308 *bHLH* loci in the soybean genome. Amino acid sequences of all *GmbHLH* genes were downloaded from the database of phytozome^2^. A comparative tree was constructed based on the amino acid sequence alignments of the 308 bHLH soybean genes (**Supplementary Table S1**). The clustering showed that there are 12 major subfamilies of related sequences that were strongly supported by bootstrap analysis; these subfamilies were titled I to XII (**Figure 1**). GmORG3 (Glyma03g28630), the red arrow-marked position, was classified as subfamily “II.” In a previous study, AtbHLH TFs were also divided into 12 subfamilies (Heim et al., 2003); the AtbHLH39, which is the orthologous gene of *GmORG3*, was classified as subfamily “Ib.” In *Arabidopsis* AtbHLH TFs were divided into 21 subfamilies in another study (Toledo-Ortiz et al., 2003); the AtbHLH39 was in the “2” subfamily.

**FIGURE 2:**
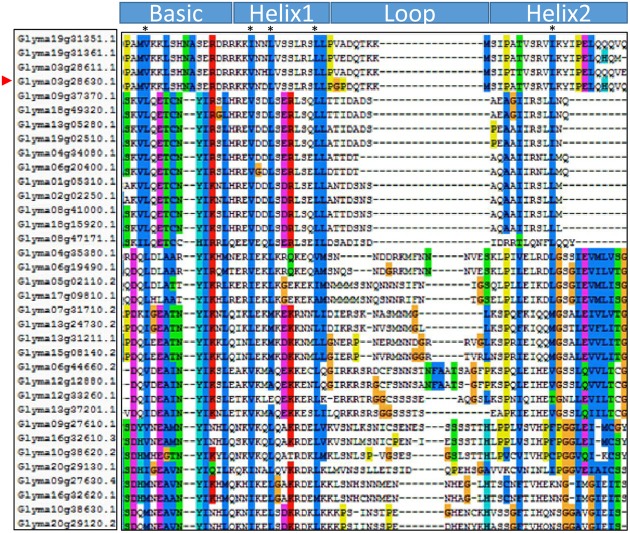
Domain structure of the GmORG3 subfamily II. Multiple sequence alignment of the bHLH domains of the 32 GmbHLH members of subfamily II. The scheme at the top depicts the locations and boundaries of the basic, helix, and loop regions within the bHLH domain. The shading of the alignment presents identical residues in different color; conserved residues contain more bases. Dots denote gaps.

The GmORG3 protein consists of 241 amino acids and contains one 61-residue-long conserved bHLH domain spanning amino acids 58–118 (**Figure 2**). The basic helix1-loop-helix2 domain sequence is PAMVKKLSHNASERDRR-KKVNDLVSSLRSLLP-GPDQTKKMS-IPATVSRVLKYIPELQH QVQ. Basic and helix domains are more conservative than are loop domains. There are conserved amino acids in the soybean bHLH transcription factors: the 4th residue in the basic part is L (Leucine)/V (Valine); the 3rd, 6th, and 13th residues in the helix1 part are V, L, and L, respectively; and 10th residue in the helix2 part is L. Those conserved amino acids should have important functions.

**FIGURE 3:**
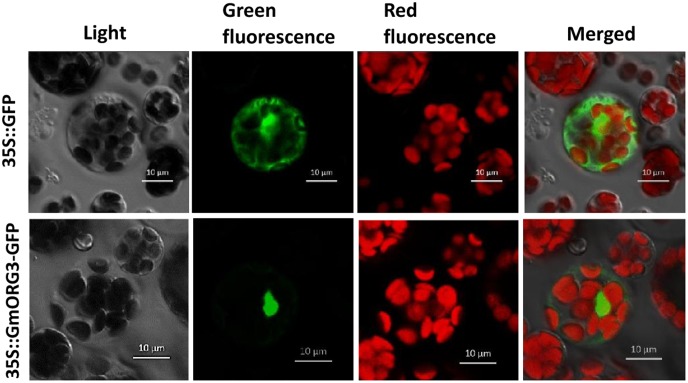
Subcellular localization of the GmOGR3 protein in *Arabidopsis* protoplasts. A construct encoding a GmORG3-green fluorescent protein fusion (35S::GmORG3-GFP) was created using the pJIT166-GFP vector. The 35S::GmORG3-GFP and GFP control plasmid (35S::GFP) were transformed into *Arabidopsis* protoplasts using PEG4000 and observed using a confocal fluorescence microscope. Nucleic acids were detected using an excitation wavelength of 350 nm and an emission wavelength of 461 nm. The GFP and chloroplast autofluorescence were detected using an excitation wavelength of 480 nm and emission wavelengths of 461 and 685 nm, respectively. Scale bars = 10 μm.

A GmORG3-green fluorescent protein (GFP) fusion construct and a control construct containing only GFP were created using the pJIT166-GFP vector. Transcription was driven by the CaMV 35S promoter in both constructs. Constructs were introduced into *Arabidopsis* protoplasts in accordance with the PEG4000-mediated method. The cells were inspected with a confocal laser-scanning microscope to visualize the subcellular localization of the proteins. The GmORG3-GFP protein was detected only in the nucleus; the control protein was observed throughout the cell (**Figure 3**). These results indicated that GmORG3 is a nuclear protein.

### Transcript Abundance of *GmORG3*

**FIGURE 4:**
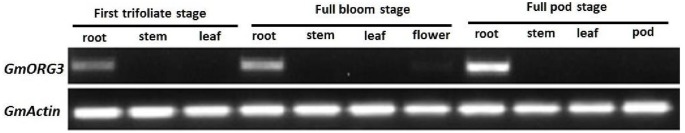
Transcript abundance of *GmORG3* in the roots, stems, leaves, flowers, and pods of soybean at the first-trifoliate, full-bloom and full-pod stages. The experiments were carried out using semi-quantitative RT-PCR; *GmActin* was used as an internal control for the normalization of cDNA templates. The PCR was repeated twice, each producing similar results.

Transcript abundance of *GmORG3* was determined in soybean plants. Total RNA was isolated from the roots, stems, leaves, flowers, and pods of soybean at the first-trifoliate, full-bloom, and full-pod stages. RNA was reverse-transcribed to obtain cDNA for semi-quantitative PCR analysis. The results showed that the transcripts of *GmORG3* were detected only in the roots at all selected stages, and the highest transcript abundance in the root tissue was detected at the full-pod stage (**Figure 4**). GmActin was used as an internal control.

**FIGURE 5:**
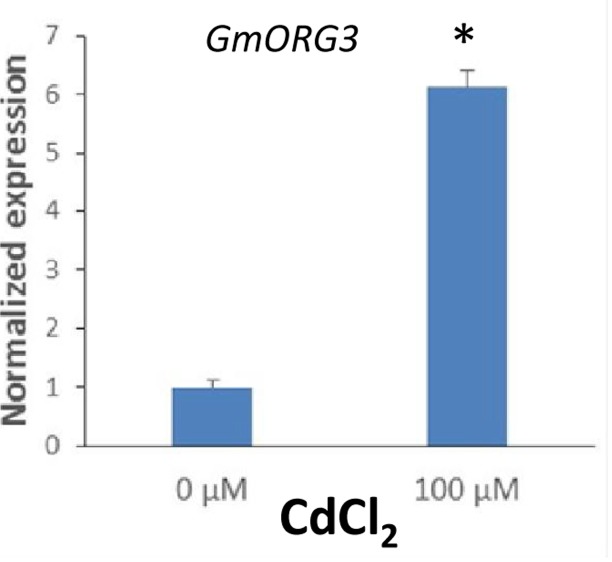
Transcript abundance of *GmORG3* under Cd exposure. Three-week-old seedlings were transferred to 100-ml glass tubes containing Hoagland solution. The tubes were divided into two groups for Cd treatments: one with 0 μM and the other with 100 μM CdCl_2_. After overnight treatment, the normalized relative transcript abundance of the *GmORG3* gene was detected in the roots using real-time quantitative RT-PCR. The *y*-axis shows RNA levels normalized to those of *GmActin*. *N* = 3, and values are the mean ± SD. ^∗^Significant difference in values at the level of *P* < 0.05 by Student’s *t*-test in comparison with those of 0 μM.

The transcript abundance response of *GmORG3* was also detected in soybean plants growing under Cd stress. qRT-PCR analyses were carried out to determine the transcript abundance patterns of the *GmORG3* gene in the roots of soybean plants. As shown in **Figure 5**, the expression of *GmORG3* was clearly up-regulated in the roots of soybean seedlings after an overnight treatment with 100 μM CdCl_2_. The expression level of *GmORG3* under CdCl_2_ was 6.13-fold higher than that under normal conditions.

## Overexpression Analysis of *GmORG3* in Yeast under Cd Stress

**FIGURE 6:**
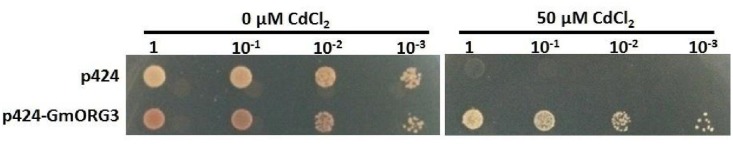
Response of *GmORG3* to cadmium tolerance in yeast. The *GmORG3* gene was fused in frame to the GAL DNA-binding domain expression vector p424 GAL and then transformed into yeast strain W303B. An empty p424 vector was used as a control. The transformants with different diluted concentrations (1, 10^-1^, 10^-2^, 10^-3^) were added to plates containing YPDA medium with either 0 or 50 μM CdCl_2_.

Heterologous expression is also an effective way to detect gene function. *GmORG3* was fused to a p424 GAL vector and overexpressed in the yeast strain WB303B. In the control YPDA medium containing 0 μM CdCl_2_, the growth rate of yeast cells expressing *GmORG3* was slightly slower than that of cells containing the empty plasmid (**Figure 6**). However, the expression of *GmORG3* significantly improved the yeast tolerance to CdCl_2_, while yeast with empty plasmids did not survive on the YPDA medium with 50 μM CdCl_2_.

### Overexpression Analysis of *GmORG3* in Soybean Mosaic Seedlings Grown under Cd Stress

The function of *GmORG3* was investigated by its construction into the plant overexpression vector pCXSN and subsequent transformation to generate soybean mosaic seedlings via *Agrobacterium rhizogenes* mediation. Soybean mosaic seedlings were tested under Cd stress to determine Cd contents in roots and leaves.

Soybean mosaic seedlings having transgenic roots and non-transgenic shoots were generated in accordance with procedures explained elsewhere. PCR-validated transgenic seedlings of *GmORG3* and those with empty vectors were grown in 100-ml glass tubes containing half-strength Hoagland solution for a few weeks to develop enough transgenic roots. Afterward, the mosaic seedlings were transferred to fresh ½-strength Hoagland solution that contained 20 μM CdCl_2_ and grown for 9 days. The leaves and roots of both seedlings with empty vector and *GmORG3* mosaic seedlings were harvested separately at 0, 1, 2, 4, 6, and 9 days after treatment. The Cd content in the samples was determined using an atomic absorption spectrometer with a continuous light source following the manufacturer’s instructions/manual (ContrAA^®^300, Analytik Jena AG).

**FIGURE 7:**
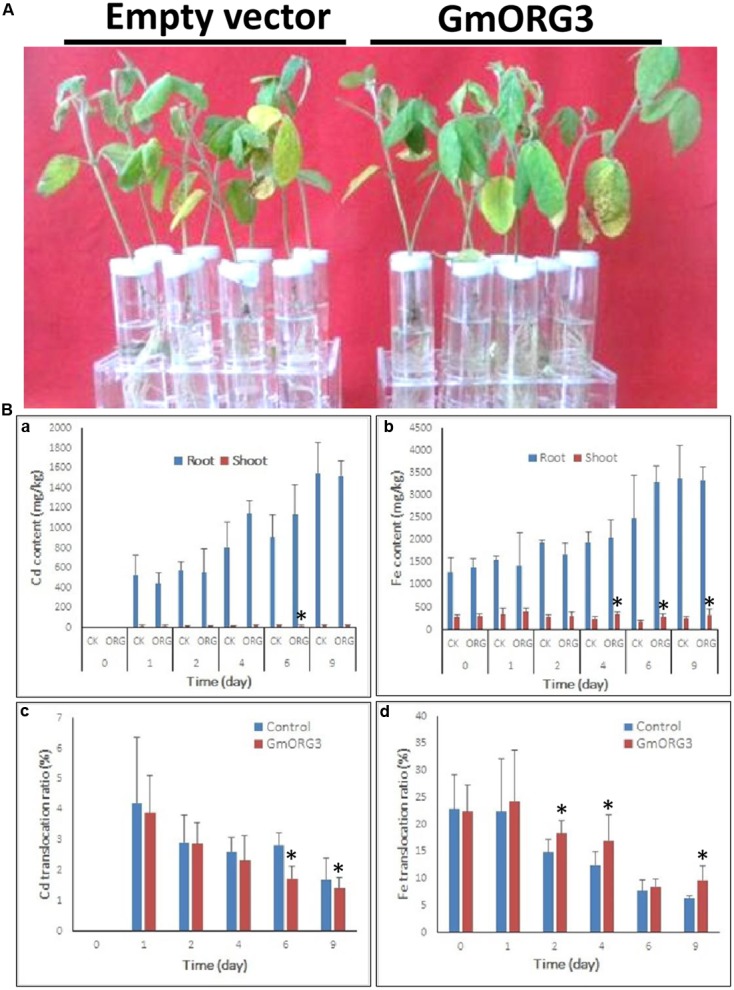
The response of mosaic soybean seedlings under Cd stress. *GmORG3* overexpression mosaic soybean seedlings were transferred to one-half-strength Hoagland solution containing 20 μM CdCl_2_ and grown for 9 days. Cd and Fe contents were determined in root and shoot samples harvested at 0, 1, 2, 4, 6, and 9 days and analyzed for their translocation ratios from roots to shoots. **(A)** Response of soybean mosaic seedlings carrying an empty vector or a *GmORG3* overexpression vector in their hairy roots grown under Cd stress. **(Ba)**, Cd content in the roots or shoots of soybean mosaic seedlings carrying an empty vector of a control check (CK) or a *GmORG3* overexpression vector. **(Bb)** Fe content in the roots or shoots of soybean mosaic seedlings carrying an empty vector of a control check (CK) or a *GmORG3* overexpression vector. **(Bc)** Cd translocation ratio from roots to shoots in soybean mosaic seedlings under Cd stress. **(Bd)** Fe translocation ratio from roots to shoots in soybean mosaic seedlings under Cd stress. ^∗^Significant difference in values at the level of *P* < 0.05 by Student’s *t*-test in comparison with those of the empty vector of the control check (CK).

Soybean mosaic plants with *GmORG3* transgenic hairy roots did not significantly differ from those with empty vector transgenic hairy roots after 9 days of growth under 20 μM CdCl_2_ stress (**Figure 7A**). However, the Cd content was lower in *GmORG3* transgenic roots than in those of plants with empty vectors during the first 2 days, but this finding was opposite at 4 and 6 days. No Cd was detected immediately after treatment (0 days) (**Figure 7Ba**). It is interesting that the translocation ratio of Cd from roots to shoots (Cd amount in shoots/total Cd amount) was consistent at every sample collection time. The *GmORG3* transgenic root seedlings had a lower ratio at all sample collection times (**Figure 7Bc**). Therefore, when roots absorb the same amount of Cd, *GmORG3* transgenic roots will translocate less Cd to shoots.

Considering that the absorption and transport of Fe is related to transporters that can also take up Cd and other metals, Fe accumulations in the roots and shoots of mosaic soybean seedlings were measured and analyzed (**Figure 7Bb,d**). The Fe content was higher in the roots than in the shoots, and a subtle increase in Fe uptake was evident with increasing time of Cd stress. *GmORG3* showed slightly lower Fe content than did empty vector seedlings during the first 2 days, whereas at 4, 6, and 9 days of treatment, *GmORG3* seedlings showed slightly higher or equal Fe content than did those with empty vectors (**Figure 7Bc**). Additionally, the Fe translocation ratio from roots to shoots (Cd amount in shoots/total Cd amount in plants) was also calculated. Interestingly, the *GmORG3* transgenic mosaic seedlings had a higher ratio at all time points (**Figure 7Bd**).

### *GmORG3* Transgenic Tobacco Growth under Cd Stress and Fe Deficiency

Since *GmORG3* significantly responded to cadmium stress and its overexpression conferred tolerance to Cd stress in yeast, further characterization of this gene was carried out. The heterologous ectopic over expression of *GmORG3* in tobacco plants showed enhanced tolerance to cadmium stress. The *GmORG3* CDS was used to prepare the construct of pCXSN-*GmORG3*, which have a 2X 35S promoter and a NOS terminator. In addition, the correct recombinant plasmid was transformed into tobacco by *Agrobacterium*-mediated leaf disk explants methods. The regenerated transformed seedlings were screened on medium containing hygromycin (100 mg/L). The putative tobacco transformants were identified using *GmORG3* gene-specific PCR analysis. The T3 seeds of transgenic plant line 1 (L-1), line 2 (L-2), and line 3 (L-3) were used in subsequent experiments.

**FIGURE 8:**
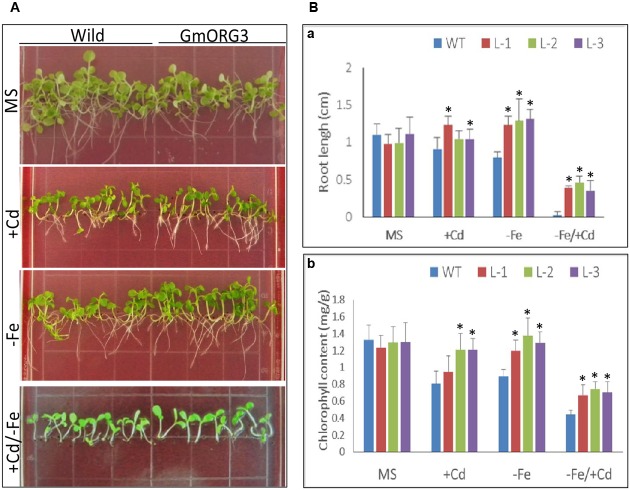
The root length and chlorophyll content of *GmORG3* transgenic tobacco plants under +Cd and/or –Fe stress. T3 *GmORG3* transgenic tobacco L-1, L-2, and L-3 seeds were grown on square plastic Petri dishes filled with one-half-strength MS (Murashige and Skoog) medium for 3 weeks. Four media treatments were established: control (MS), MS medium with 20 μM Cd (MS/+Cd), MS medium without Fe (MS/–Fe), and MS medium with 20 μM Cd but without iron (MS/+Cd-Fe). **(A,Ba)**, Root length of transgenic or wild-type tobacco seedlings on four different media treatments. **(Bb)**, Chlorophyll content in fresh seedlings on four different media treatments. ^∗^Significant difference in values at the level of *P* < 0.05 by Student’s *t*-test in comparison with those of the wild-type (WT) seedlings.

Wild-type (WT) and transgenic tobacco L-1, L-2, and L-3 seeds were sown on ^1^/_2_ MS medium (MS), ^1^/_2_ MS medium supplemented with 20 μM CdCl_2_ (MS/+Cd), ^1^/_2_ MS medium without Fe (MS/-Fe) and ^1^/_2_ MS medium with +Cd/-Fe (MS/+Cd-Fe) (**Figure 8**). The growth of transgenic seedlings on MS control medium was similar to that of WT seedlings (**Figure 8A**): the roots of transgenic plants were slightly shorter than those of WT. The root length assays indicated that the average root length of WT plants was 1.10 cm and that of transgenic plants L-1, L-2, and L-3 were 0.98, 0.99, and 1.11 cm, respectively (**Figure 8Ba**). The leaf color of both WT and transgenic plants was also similar. The content of chlorophyll was 1.30 mg/g of fresh weight (FW) in the leaves of WT and was 1.24, 1.30, and 1.30 mg/g (FW) in the leaves of transgenic lines L-1, L-2, and L-3, respectively (**Figure 8Bb**).

The growth of transgenic seedlings on MS medium supplemented with 20 μM CaCl_2_ showed obvious differences with that of WT. The roots of transgenic seedlings were longer than those of WT; there were significant differences between root lengths of transgenic L-1 and WT seedlings but no significant differences between those of transgenic L-2 and L-3 with WT seedlings (**Figure 8Ba**). The chlorophyll contents varied significantly between all transgenic lines and WT (**Figure 8Bb**). On Fe-deficient medium, the root lengths of transgenic seedlings were significantly longer than those of WT, while chlorophyll contents in transgenic lines were higher than those of WT (**Figure 8B**). The responses of all transgenic and WT seedlings were worse on +Cd/-Fe medium than on other media; all seedlings grown on +Cd/-Fe medium were short. However, the chlorophyll content of all the transgenic lines was significantly (*P* < 0.05) higher than that of WT. Similarly, the root length of transgenic seedlings was also significantly (*P* < 0.01) longer than that of WT.

The above results demonstrated that overexpressing *GmORG3* improves the tolerance of transgenic tobacco plants to cadmium stress.

## Discussion

Basic/helix-loop-helix transcription factors, which are common types of transcription factors, is one of the largest gene families in plants with important biological functions (Pires and Dolan, 2010). In *Arabidopsis*, 162 bHLH-encoding genes have been identified, which indicate that bHLH transcription factors constitute the second largest transcription factor family after the MYB superfamily, which has 190 members (Riechmann et al., 2000; Dubos et al., 2010). Several studies have reported the existence of this superfamily in other species, such as rice (*Oryza sativa* L.), which has 180 bHLH transcription factors; tobacco (*Nicotiana tabacum* L.), which has 190; and grape (*Vitis vinifera* L.), which has more than 191 (Xiong et al., 2005; Jaillon et al., 2007; Rushton et al., 2008). We isolated 308 bHLH transcription factors in soybean (**Figure 1**), which may be the largest transcription factor family in the whole genome reported thus far. The members of this superfamily are involved in many biological processes such as stress responses (Osorio et al., 2012), nodule growth and NH_4_^+^ transport (Chiasson et al., 2014). In our study, the GmbHLHs were divided into 12 subfamilies (**Figure 1**); previous studies in other species reported six subfamilies in the animal genomes (Atchley and Fitch, 1997), 21 subfamilies in *Arabidopsis* (Toledo-Ortiz et al., 2003), and 22 subfamilies in rice (Li et al., 2006). This suggests that the classification of bHLH transcription factors in plants is more complex than in animals, and more research is needed to classify plant bHLH transcription factors.

GmORG3 belongs to the bHLH transcription factor superfamily. GmORG3 contains one 61-amino acid domain that comprises basic-helix1-loop-helix2 domains (**Figure 2**). It is interesting that the basic and helix domains were more conserved than the loop domain was. The basic region binds with the DNA E-box motif (5′-CANNTG-3′) (Toledo-Ortiz et al., 2003; Li et al., 2006). The helix domain needs stable hydrophobic amino acids such as the position 4 L (Leucine)/V (Valine) in helix1; the position 3 V, 6 L and 13 L in helix2; and the position 10 L in the bHLH structure to form homo- or heterodimers (Ferré-D’Amaré et al., 1993). Therefore, the results of the previous studies and our results suggest that GmORG3 may interact with itself or other GmbHLHs before binding with the E-box in the promoter, but this speculation needs more evidence.

BLASTing the *Arabidopsis* genome showed that the *GmORG3* orthologous gene was the bHLH gene *AtbHLH39* (*AtORG3*), which is expressed during both Fe deficiency stress (Yuan et al., 2008) and Cd stress (Wu et al., 2012). Studies have also shown that AtbHLH39 interacts with AtbHLH29 in the nucleus. The overexpression of AtbHLH39 with AtbHLH29 in *Arabidopsis* seedlings altered the expression pattern of the Fe uptake genes FRO2 (ferric chelate reductase 2) and IRT1 (iron-regulated transporter 1) from being induced to constitutive and enhanced seedling tolerance to Cd. The determination of the Fe and Cd content showed that co-overexpression of AtbHLH39 and AtbHLH29 increased Cd sequestration in roots and improved Fe homeostasis of shoots (Yuan et al., 2008; Wu et al., 2012). Based on our results, GmORG3 was also localized in the nucleus (**Figure 3**) and was significantly up-regulated by Cd stress (**Figure 5**). This suggests that the structure of similar genes may have a similar function. However, GmORG3 was only expressed in the roots during all growth stages (**Figure 4**). This may suggest that *GmORG3* is important in the translocation of metals from the roots to shoots. The overexpression of *GmORG3* in yeast W303B cells showed improved growth compared with that of the control on CdCl_2_-supplemented medium (**Figure 6**), which suggests a similar function of *GmORG3* in plants. In the present study, we chose tobacco plants as the heterologous expression material for our phytoremediation study because of its fast growth, high biomass productivity without special care, and overall significance (Evangelou et al., 2007). *GmORG3* transgenic tobacco lines were developed using *Agrobacterium*-mediated transformation. The T3 seeds of tobacco lines L-1, L-2, and L-3 were sown on MS medium with 20 μM CdCl_2_ and subjected to exogenous Cd stress. The transgenic tobacco plants showed enhanced Cd tolerance via significant increases in growth, root length, and chlorophyll content compared to those of wild-type controls (**Figure 8**). The Cd detoxification in plants is a very intricate proocess, involving a number of different physiological and molecular mechanisms, such as reduced uptaking from soil, binding in cell wall, enrichment in the epidermal hairs, chelation with phytochelatins and reduced translocation from roots to shoots. Plant phenotypes exhibiting tolerance usually cannot be explained simply by a single mechanism. Detoxification usually is a result of multiple mechanisms joint action. Several studies have indicated that Cd stress can hinder chlorophyll biosynthesis (Küpper et al., 2007; Redondo-Gómez et al., 2009; Rai et al., 2013; Çelekli et al., 2015; Li et al., 2015; Cheng et al., 2016). In the present study, *GmORG3* reduced the damage of Cd stress in the transgenic tobacco plants and increased the chlorophyll content. This suggests that *GmORG3* might have increased the stability of the photosynthesis system. However, the mechanism by which *GmORG3* stabilizes the photosynthetic system is not clear; here, the mosaic soybean seedling experiments could provide the results (**Figure 7**). In conclusion, *GmORG3* exhibited lower Cd translocation ratios from roots to shoots in mosaic soybean seedlings, and the results were in accordance with those of Wu et al. (2012). Recently, a major soybean QTL (quantitative trait locus) associated with low Cd concentrations in seeds was identified (Jegadeesan et al., 2010). This QTL accounted for 57.3% of the phenotypic variation. In this QTL, a gene for the plasma membrane H^+^-ATPase was found to be located at the locus, and this gene may be the key gene *Cda1* (low Cd accumulation). Therefore, *Cda1* could be the target gene of GmORG3. Future experiments should study the interaction between those two genes.

## Materials and Methods

### Plant Materials and Growth Conditions

Plants of the Chinese cultivar Dongnong 690 of soybean (*Glycine max* L. Merr.), the Columbia ecotype of *Arabidopsis* (*Arabidopsis thaliana*) and tobacco (*Nicotiana tabacum* L.) were used in this study. Dongnong 690 plants was sown in 20-cm pots filled with a 1:1 mixture of vermiculite and nutrient-rich soil that were placed in the greenhouse. *Arabidopsis* seeds were sown in small pots containing vermiculite with sufficient water; the pots were covered with plastic wrap and placed into an incubator. Photoperiod and temperature were 12 h of light at 22°C and 12 h of darkness at 20°C. Seeds of tobacco and transgenic lines were sterilized with 10% NaClO for 10 min, then washed more than three times with double distilled water. After vernalization at 4°C in the dark for 2 days, the seeds were sown onto plates with 1/2 MS medium at pH 5.8. The plates were incubated at 22°C under a 12-h photoperiod. All the seedlings were then used for further analysis.

### Sequence and Domain Analysis of GmORG3

Bioinformatics analyses were performed by various web-based software programs. The amino acid sequences of the soybean bHLH transcription factors including GmORG3 were downloaded from the Phytozome database^2^ and aligned using Clustal Omega web software^3^. Other bioinformatics software programs utilized included MEGA 5 and PowerPoint 10 for general sequence homology analysis, drawing of figures, domain analyses and the prediction of similarity and identity among different members of the subfamilies.

### Semi-quantitative RT-PCR and Quantitative PCR

Total RNA was isolated from the tissues of roots, stems, leaves, flowers, and pods of Dongnong 690 at the first-trifoliate, full-bloom and full-pod stages using an SV total RNA kit (Promega, United States). RNA samples were quantified using a Nanodrop spectrophotometer (United States). cDNA was synthesized from 5 μg of total RNA with a SuperScript RT III first-strand cDNA synthesis kit (Invitrogen, San Diego, CA, United States) according to the manufacturer’s instructions. The synthesized cDNA was used as PCR template. GmActin was used as an internal control for semi-quantitative RT-PCR analysis. The GmORG3-specific primer pair, ORG3-F (5′-CGAGATCATCATAGCATTATCT-3′) and ORG3-R (5′-CTCCATTCTAATTTCCGAACT-3′), was utilized for expression analysis (semi-qPCR). The *GmActin* (accession number: NM_001250673) primer pair, GmActin-F (5′-CGGTGGTTCTATCTTGGCATC-3′) and GmActin-R (5′-GTCTTTCGCTTCAATAACCCTA-3′) was used as an internal control. PCR was carried out in 1x PCR buffer supplemented with dNTPs at 150 μM, 1.5 U of Taq DNA polymerase and 5 pmol of each gene-specific primer according to the following program: initial denaturation at 95°C for 5 min; 27 cycles of 94°C for 30 s, 55°C for 30 s and 72°C for 30 s; and a final extension step at 72°C for 5 min. Semi-quantitative RT-PCR experiments were repeated three times. The amplification products were separated via agarose gel electrophoresis and analyzed using a fluorescence imaging system (Peiqing CN).

Total RNA from the roots of 3-week-old soybean seedlings grown under control and 50 μM CdCl_2_ stress conditions for 12 h was extracted, reverse-transcribed and used for real-time quantitative PCR analysis. Sample preparation and PCR analysis were performed using the SYBR Premix Ex Taq in a Roche Light Cycler 2.0 with Light Cycler software (build 4.1.1.21) (LightCycler Carousel-based system, F. Hoffmann-La Roche Ltd, Germany). A 10-μl reaction consisted of 5 μl of SYBR Premix Ex Taq, 0.8 μl of each primer (forward and reverse, 10 mM), 1 μl of template and 2.4 μl of ddH_2_O. The GmORG3-specific primer pair, qORG-F (5′-TCGTTCACTTCTTCCTGGGC-3′) and qORG-R (5′-ATAGTGGAGCCTTGTGAGCC-3′), was utilized for its expression study under control and Cd stress conditions. Forward (5′-CGGTGGTTCTATCTTGGCATC-3′) and reverse (5′-GTCTTTCGCTTCAATAACCCTA-3′) primers of the soybean GmActin gene were used as an internal control for the expression analysis of GmORG3. An equal amount of cDNA template was used for each sample reaction, including the internal control. qPCR amplification conditions included an initial denaturation for 30 s at 95°C followed by 35 quantification cycles consisting of denaturation for 5 s at 94°C, annealing for 10 s at 58°C, and extension for 30 s at 72°C. A melting curve analysis was then performed to confirm the specificity of the PCR products. The real-time quantitative PCR analysis was repeated in three independent experiments.

### *GmORG3* Mosaic Soybean Seedlings

An *Agrobacterium rhizogenes*-mediated soybean genetic transformation system was used to introduce the *GmORG3* overexpression cassette into the hairy roots of soybean variety Dongnong 690. Soybean seeds were sown into pots containing vermiculite and nutrient-rich soil in a 1:1 ratio. Upon the emergence of cotyledons, healthy and robust seedlings were selected for transformation. Agrobacteria carrying the *GmORG3* overexpression cassette/plant expression vector pCXSN-GmOGR3 were streaked onto an LB plate containing kanamycin and precultured at 28°C for 2 days. A single colony was used to inoculate liquid LB medium that contained kanamycin as a selectable marker. The culture was grown at 28°C for 12 h with shaking at 200 rpm. The culture was centrifuged at 4500 rpm for two min at room temperature to harvest the bacteria. The pellet was twice suspended gently in a 10 mM MgCl_2_ solution. The OD_600_ of the final bacterial suspension was adjusted to 0.4–0.6. Young seedlings with unfolded cotyledons were infected at the cotyledonary node and/or the hypocotyl with *Agrobacterium rhizogenes* carrying the pCXSN-*GmORG3* construct. The injection point was covered immediately after inoculation with a wet vermiculite/soil mixture. The infection sites were kept in a high-humidity environment. Water was applied as required. The seedlings were grown in a greenhouse at 25°C with a 12-h photoperiod and approximately 60% relative humidity. Hairy roots were induced at the injection point after 1 week; the roots grew to 2–3 cm in length 1 week after inoculation. As such, the hairy roots developed 3 weeks after sowing. The main roots were removed when the emerged hairy roots could support the plants. Mosaic seedlings with one hairy root were transferred to 100-ml glass tubes containing ½-strength Hoagland solution. The glass tubes containing mosaic soybean seedlings were placed in a growth chamber with 25/22°C day/night temperature and a 12-h photoperiod for approximately 1 week. The Hoagland solution in the glass tubes was replaced every other day. Once the hairy roots reached bottom of the tube, the transformation of hairy roots was verified using PCR analysis. PCR was performed using a CaMV35S promoter-specific forward primer (5′-CAATCCCACTATCCTTCGCAAGACC-3′) and a *GmORG3* gene-specific reverse primer. Genomic DNA from the hairy roots was used as a template. One set of soybean mosaic seedlings carrying a pCXSN empty vector was prepared for use as a control. Transgenic hairy roots of mosaic seedlings were immersed in ½-strength Hoagland solution containing 20 μM CdCl_2_ and grown for 9 days in a growth chamber. The Hoagland solution containing Cd was replaced every other day. At 0, 1, 2, 4, 6, and 9 days, roots and shoots tissues were harvested for analysis of Cd and Fe content. The experiment was repeated three times.

### *GmORG3*-Transformed Tobacco

For the heterologous expression of the *GmORG3* in tobacco, *A. tumefaciens* strain EHA105 containing the pCXSN:2xCaMV35S::*GmORG3*-NOS construct was used to get transgenic tobacco plant according to a reported protocol, with a little modification. The hygromycin were used as selection maker. The positive transformed tobacco seedlings were grown in a contained net house. When the plantlets were large enough, DNA was extracted from the leaves, and positive plantlets were screened using PCR. T1 seeds were then harvested, and selection continued until the T3-generation seeds were collected and cultured for further study.

### Subcellular Localization

*Arabidopsis* protoplasts were isolated as previously described (Yoo et al., 2007). The CDS of *GmORG3* was inserted into the pJIT166-GFP vector without a termination codon to create an in-frame fusion between *GmORG3* and the green fluorescent protein. The fusion construct (p35S::GmORG3-GFP) and a control construct (p35S::GFP) were introduced into *Arabidopsis* protoplasts using the PEG4000 method as described by Abel and Theologis (1994). After the incubation of transformed *Arabidopsis* protoplasts in culture solution for 18–24 h without light at room temperature, imaging was performed using a confocal microscope (Zeiss, LSM510 Meta, Carl Zeiss AG) and a 40X lens. The fluorescence of GFP was measured at 495–545 nm, and chloroplast autofluorescence was measured at 480–685 nm.

### Determination of Cd, Fe, and Chlorophyll Contents

Regarding the experiments to determine the function of *GmORG3* under Cd toxicity stress, mosaic soybean seedlings were generated following the procedure described in the section “Development and treatment of *GmORG3* mosaic soybean seedlings” above. Root and shoot tissue was harvested, and the Cd and Fe contents of the samples were then determined. Samples were kept at 105°C for 20 min and dried at 80°C to constant weight. Dry tissues were ground into a powder, and 1 ml of 100 mM acetic acid was added. Samples were incubated at 90°C for 6 h. Cadmium and iron contents were then determined using an atomic absorption spectrometer with a continuous light source (ContrAA^®^300, Analytik Jena AG). The analysis was repeated three times.

Tobacco seeds of the *GmORG3* transgenic lines and wild-type plants were grown on square plastic Petri dishes filled with 1/2 MS medium with 20 μM CdCl_2_, without Fe, and 1/2 MS medium supplemented with 20 μM Cd but without Fe. The dishes were placed into a growth chamber. Three weeks later, the seedlings were imaged, chlorophyll contents were determined, root lengths were measured and samples were harvested. The estimation of chlorophyll from seedlings was performed in accordance with the method of Arnon (1949): 100 mg of tissue was homogenized thoroughly in 1 ml of 80% acetone and centrifuged at 3000 rpm for 2–3 min. The supernatant was retained, and absorbance was measured using a spectrophotometer at 663 and 645 nm. Total chlorophyll was estimated using the following equation: total chlorophyll = [(20.2 × A_645_ + 8.02 × A_663_)/(1000 × W)] × V. The experiment was repeated three times.

## Author Contributions

ZX, XL, HM, and HS conceived, designed and conducted the experiments. XH, LX, YH, DZ, and BT analyzed the data and results. ZX wrote the manuscript. HS and HM monitored the experiments and critically commented on the manuscript. All authors read and approved the final manuscript.

## Conflict of Interest Statement

The authors declare that the research was conducted in the absence of any commercial or financial relationships that could be construed as a potential conflict of interest.

## Footnotes

1 http://www.harvestplus.org/what-we-do/nutrition

2 https://phytozome.jgi.doe.gov/pz/portal.html

3 http://www.ebi.ac.uk/Tools/msa/clustalo/

## Supplementary Material

TABLE S1 A comparative tree based on the amino acid sequence alignments of the 308 bHLH soybean genes.
